# Pancreatic exocrine insufficiency in patients with chronic heart failure and its possible association with appetite loss

**DOI:** 10.1371/journal.pone.0187804

**Published:** 2017-11-20

**Authors:** Tingting Xia, Xichen Chai, Jiaqing Shen

**Affiliations:** 1 Department of Gastroenterology, The First Affiliated Hospital of Soochow University, Canglang District, Suzhou, Jiangsu Province, China; 2 Department of Cardiology, The First Affiliated Hospital of Soochow University, Canglang District, Suzhou, Jiangsu Province, China; Hospital Universitario de la Princesa, SPAIN

## Abstract

**Background:**

Appetite loss is one complication of chronic heart failure (CHF), and its association with pancreatic exocrine insufficiency (PEI) is not well investigated in CHF.

**Aim:**

We attempted to detect the association between PEI and CHF-induced appetite.

**Methods:**

Patients with CHF were enrolled, and body mass index (BMI), left ventricular ejection fraction (LVEF), New York Heart Association (NYHA) cardiac function grading, B-type natriuretic peptide (BNP), serum albumin, pro-albumin and hemoglobin were evaluated. The pancreatic exocrine function was measured by fecal elastase-1 (FE-1) levels in the enrolled patients. Appetite assessment was tested by completing the simplified nutritional appetite questionnaire (SNAQ). The improvement of appetite loss by supplemented pancreatic enzymes was also researched in this study.

**Results:**

The decrease of FE-1 levels was found in patients with CHF, as well as SNAQ scores. A positive correlation was observed between SNAQ scores and FE-1 levels (r = 0.694, p < 0.001). Pancreatic enzymes supplement could attenuate the decrease of SNAQ scores in CHF patients with FE-1 levels <200 μg/g stool and SNAQ < 14.

**Conclusions:**

Appetite loss is commonly seen in CHF, and is partially associated with pancreatic exocrine insufficiency. Oral pancreatic enzyme replacement therapy attenuates the chronic heart failure-induced appetite loss. These results suggest a possible pancreatic-cardiac relationship in chronic heart failure, and further experiment is needed for clarifying the possible mechanisms.

## Introduction

Often caused by myocardial infarction, hypertension, valvular heart diseases, cardiomyopathy and other forms of chronic artery diseases, chronic heart failure (CHF) isa progressive and irreversible disease, which is commonly seen in the department of cardiology [1, 2]. The treatment of CHF still depends on the severity and cause, and is often symptom relieving, though the pathogenesis of this disease is well investigated, which leads to a high rate of morbidity and a potentially dead condition [3–5]. Unplanned readmission rates after hospitalization remain high, despite improvements in outcome with medicaland device therapy [6–9].

Appetite loss is one complication of CHF and induces a lack of intake in energy, protein and other nutrients, leading to malnutrition [10]. Malnutrition is highly prevalent in patients with CHF and also is an important riskfactor for morbidity and mortality [11]. Ischemia and congestion in peripheral tissues caused by heart failure may result in function loss in many organs such as kidney, liver, stomach, intestine, which may be a possible reason of appetite loss in this disease [12]. A recent study show that pancreatic fecal elastase-1 (FE-1) levels decrease significantly in patients with acute decompensated heart failure, indicating that pancreatic exocrine insufficiency (PEI) may also contribute to the determination of malnutrition in heart failure [13], but the relationship between PEI and appetite loss is unclarifed.

In this study, the pancreatic exocrine function was measured by FE-1 levels in the enrolled patients of CHF. Appetite assessment was tested by the simplified nutritional appetite questionnaire (SNAQ). The association between FE-1 and CHF-induced appetite loss was also investigated. The improvement of appetite loss by supplemented pancreatic enzymes was researched at the same time in this study.

## Materials and methods

### Patients

In this study, patients with CHF were enrolled, who were hospitalized and followed up in the Department of Cardiology of the First Affiliated Hospital of Soochow University, between August 2013 and December 2014. The diagnosis of CHF was based on the presence of current or previous symptoms, characteristic clinical signs, and evidence of ventricular dysfunction, according to the criteria of the American College of Cardiology/American Heart Association 2005 Guideline Update for the Diagnosis and Management of heart failure [14]. The severity of heart failure was categorized by NYHA (New York Heart Association) functional class criteria. The inclusion criteria in this study were: (1) age ≥ 18 years; (2) a documented history of heart failure of ≥ 6 months; (3) left ventricular ejection fraction (LVEF) ≤ 45% as assessed by echocardiography (performed at admission using Simpson’s planimetric method); (4) clinical stability and unchanged heart failure medications for ≥ 1 month. Exclusion criteria included: (1) acute coronary and/or coronary revascularization within 3 months preceding the study; (2) pancreatic diseases, including acute pancreatitis, chronic pancreatitis and pancreatic cancer; (3) renal dysfunction (serum creatinine > 177 μmol/L); (4) liver dysfunction; (5) acute infection, or a history of malignancy.

The healthy persons in the control group had no systemic diseases or symptoms, and were drawn from outside the hospital. Written informed consent was given to all the patients before participation and the study was approved by the ethics committee of the First Affiliated Hospital of Soochow University and complied with the Declaration of Helsinki.

### Experimental design

In experiment 1, blood and feces samples were collected in the enrolled patients (N = 104) with CHF. Blood samples were used for analyzing heart failure-related parameters. Feces were used for the measurement of FE-1 levels. Appetite assessment was tested by SNAQ. In experiment 2, the effect of pancreatin on the appetite loss was evaluated in CHF patients with FE-1 levels <200 μg/g stool and SNAQ < 14 (N = 60). Pancreatin is also known as pancreatic enzymes (the commercial mixtures of amylase, lipase, and protease), which are used to treat malabsorption syndrome due to pancreatic problems. In this randomized controlled trial, patients were randomly allocated to one of two groups (placebo group and treatment group) using a computer-generated table [15]. Patients in placebo group received regular treatment for heart failure plus placebo (N = 30). Patients in treatment group received regular treatment for heart failure plus pancreatin (Creon 10000^®^, 2^#^, po, t.i.d.) (N = 30).

### Laboratory measurements

Following an overnight fast (8 hours), blood samples were collected. Venous blood was drawn and sera were then collected after centrifugation at 3000×g for 15 min after coagulation. Serum was stored in the refrigerator at 4°C in aliquots until analysis. All laboratory variables were measured by a central laboratory that undergoes regular internal and external quality audits. A fully automatic biochemical analyzer (Roche, Mannheim, Germany) was used to determine the serum parameters. N-terminal pro-B type natriuretic peptide (NT-proBNP) was measured by using an immunoassay based on electrochemiluminescence with the Elecsys 1010/2010 System (Roche Diagnostics, Mannheim, Germany).

### Serum TNF-α, IL-1β, IL-6 and leptin measurement

For quantitative determination of human TNF-α, IL-1β, IL-6 and leptin in **se**rum, a commercially available ELISA kit was applied according to the manufacturer’s instructions (R&D, MN, USA). This assay kits employ the quantitative sandwich enzyme immunoassay technique and antibodies specific for TNF-α, IL-1β, IL-6 and leptin have been pre-coated onto a microplate with the high sensitivity and excellent specificity for detection. No significant cross-reactivity or interference between human TNF-α, IL-1β, IL-6 and leptin and analogues was observed.

### Assessment of pancreatic exocrine insufficiency

Pancreatic exocrine function was evaluated by FE-1 levels as mentioned previously. FE-1 levels were measured by a commercially available Enzyme-Linked Immunosorbent Assay kit (Immundiagnostik, Bensheim, Germany) according to manufacturer’s instructions. FE-1 level ≥200 μg/g stool indicated normal pancreatic function, whereas the concentration of 100 to 200 μg/g stool indicated mild to moderate PEI, and FE-1 <100 μg/g stool were qualified as severe PEI.

### Appetite assessment

When at admission, all enrolled patients were asked to complete the simplified nutritional appetite questionnaire (SNAQ) [16]. Subjects were asked to complete the questionnaire by circling the correct answers and then tally the results based upon the following numerical scale: a = 1, b = 2, c = 3, d = 4, e = 5. The sum of the scores for the individual items constitutes the SNAQ score. SNAQ is a 4-item single-domain questionnaire and the 4 items are as follows: 1. My appetite is: a. very poor, b. poor, c. average, d. good, e. very good; 2. When I eat: a. I feel full after eating only a few mouthfuls, b. I feel full after eating about a third of a meal, c. I feel full after eating over half a meal, d. I feel full after eating most of the meal, e. I hardly ever feel full; 3. Food tastes: a. very bad, b. bad, c. average, d. good, e. very good; 4. Normally I eat: a. less than one meal a day, b. one meal a day, c. two meals a day, d. three meals a day, e. more than three meals a day.

### Statistical analysis

Statistical analysis was performed using SPSS software version 18.0 (Chicago, IL). The data are expressed as means ± SD. One-way ANONA was used for comparing data with a normal distribution and a Kruskal-Wallis test was used for non-parametric data. Pearson’s χ^2^ test or Fisher’s exact test was used for comparison of categorical variables. Spearman coefficient was used for measuring linear correlation between variables. Values of *P*< 0.05 were considered statistically significant.

## Results

**Fig 1:**
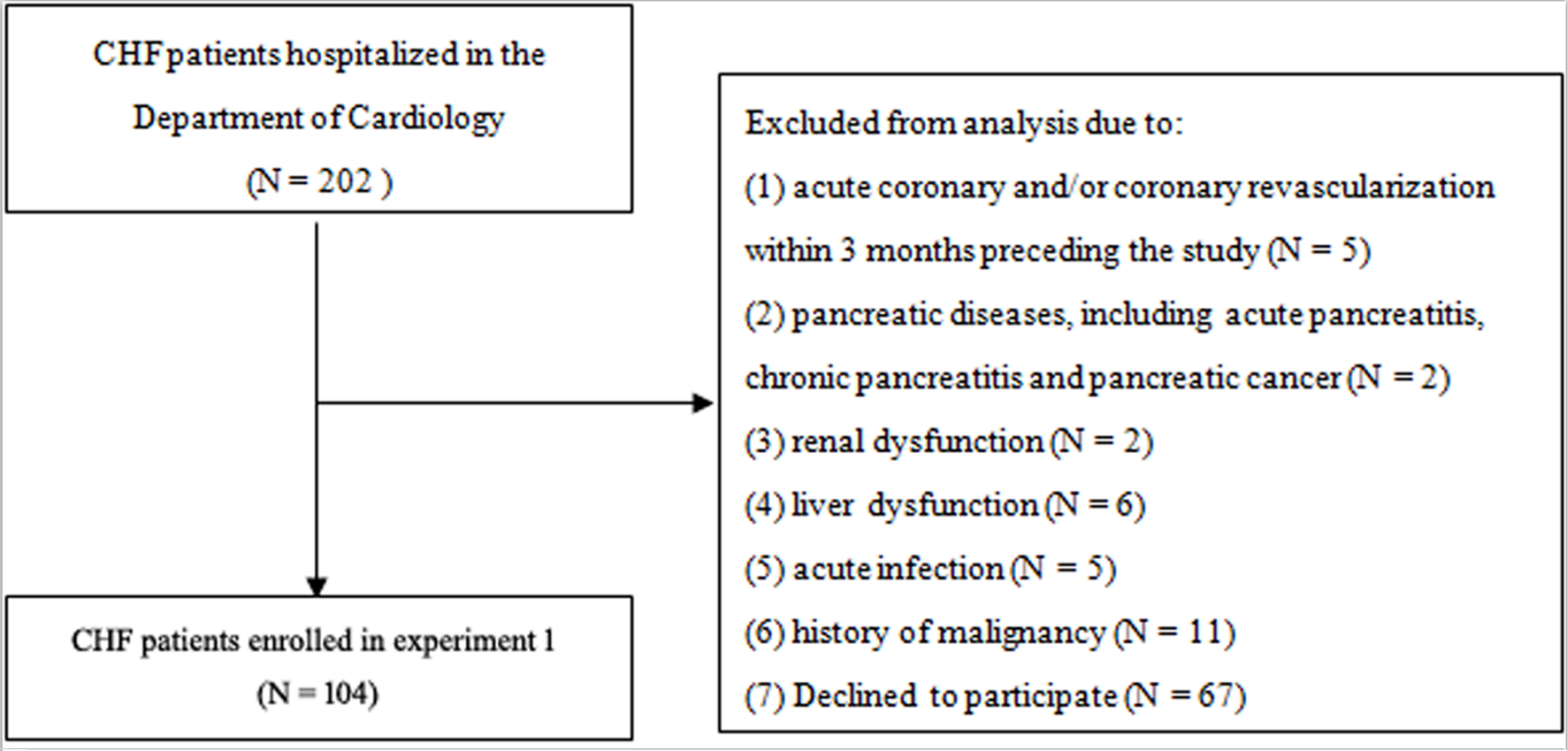
Flow diagram of patients selection in experiment 1.

A hundred and four patients with CHF were enrolled in experiment 1, with 20 healthy persons served as control (Fig 1). Patients are divided into three groups according to NYHA score (NYHA I/II N = 32; NYHA III N = 42; NYHA IV N = 30). The comparison of demographic data among the control and patient groups are shown in Table 1. Among the patient groups, there were no significant differences in male/female distribution, mean age, BMI, HF causes, systolic and diastolic blood pressure and heart rate, as well as the incidence of diabetes, smoking and alcohol consumption. The frequency of gastrointestinal symptoms, such as abdominal pain, weight loss, bloating and diarrhea was similar among the patients with different NYHA score. Compared with the control group, a significant reduction were found in patient groups in ejection fraction (EF), total protein, albumin and pro-albumin, while NT-proBNP had an opposite tendency. A reduction of hemoglobin was also found in patients with CHF, but there is no significant difference among the patient groups. Serum creatinine, blood urea nitrogen, TNF-α, IL-1β and IL-6were increased along with the NYHA grading, while leptin showed the opposite.

**Table 1:** The comparison of demographic data among the control and patients with CHF.

	Control (n = 20)	NYHA I/II (n = 32)	NYHA III (n = 42)	NYHA IV (n = 30)
Men, n (%)	12 (60.00)	32 (61.54)	30 (57.69)	27 (67.50)
Age (y)	71.50±8.58	71.33±9.10	69.81±8.06	70.02±6.31
BMI, kg/m^2^	22.25±3.11	21.15±2.93	21.92±2.73	22.52±3.20
CHF causes, n (%)				
Hypertensive heart diseases	-	15	17	16
Ischemic heart diseases	-	10	13	13
Dilated cardiomyopathy	-	3	7	6
Valvular diseases	-	2	4	3
Others	-	2	1	2
SBP (mmHg)	128.75±2.35	129.00±2.41	127.83±5.33	127.71±2.78
DBP (mmHg)	78.45±3.76	79.13±3.31	77.52±3.43	80.59±2.41
Heart rate (bpm)	79.28±8.35	82.63±7.62	88.59±7.10	84.13±2.69
Diabetes, n (%)	0	6	8	6
Smoking, n (%)	0	9	13	8
Alcohol, n (%)	0	6	10	5
Symptoms				
Abdominal pain	0	0	2	2
Weight loss	0	3	4	3
Bloating	0	2	5	3
Diarrhea	0	0	0	0
Ejection fraction (%)	64.17±7.61	47.11±11.45^a^	43.47±14.15^a^^,^^b^	39.99±11.60^a^^,^^b^^,^^c^
NT-proBNP (pg/ml)	36.27±8.93	1174.87±297.36^a^	1315.53±309.54^a^^,^^b^	1400.20±557.21^a^^,^^b^^,^^c^
Total protein (g/L)	63.57±6.26	63.39±7.71	61.53±7.12^a^^,^^b^	58.92±8.33^a^^,^^b^^,^^c^
Albumin (g/L)	49.82±5.11	42.15±4.37^a^	36.38±7.55^a^^,^^b^	31.53±6.73^a^^,^^b^^,^^c^
Pro-albumin (mg/L)	326.20±27.12	195.73±42.56^a^	142.97±51.29^a^^,^^b^	122.36±36.75^a^^,^^b^^,^^c^
Hemoglobin (g/L)	135.71±12.28	123.57±12.67	110.92±14.32	112.36±16.95
sCr (μmol/L)	44.84±11.96	53.26±13.67	58.14±15.49^a^	87.55±18.76^a^^,^^b^^,^^c^
BUN (mmol/L)	3.98±0.93	4.77±1.32	6.92±2.06^a^^,^^b^	8.15±2.68^a^^,^^b^^,^^c^
TNF-α (pg/mL)	96.67±17.48	121.85±36.30	204.08±68.81^a^^,^^b^	282.07±71.80^a^^,^^b^^,^^c^
IL-1β (pg/mL)	20.84±14.39	37.48±15.97^a^	47.64±21.73^a^^,^^b^	76.72±21.78^a^^,^^b^^,^^c^
IL-6 (pg/mL)	4.54±3.18	8.87±3.40^a^	10.87±4.52^a^	13.32±5.93^a^^,^^b^^,^^c^
Leptin (ng/mL)	10.97±2.35	9.61±2.75	7.09±2.46^a^^,^^b^	5.33±2.90^a^^,^^b^^,^^c^

CHF: chronic heart failure; NYHA:New York Heart Association; BMI: body mass index; SBP: systolic blood pressure; DBP: diastolic blood pressure; NT-proBNP: N-terminal pro-B type natriuretic peptide; sCr: serum creatinine; BUN: blood urea nitrogen; TNF-α: tumor necrosis factor-α; IL-1β: interleukin-1β; IL-6: interleukin-6. Values expressed in mean±SD or frequency

^a^*P*<0.05: versus control

^b^*P*<0.05: versus NYHA I/II

^c^*P*<0.05: versus NYHA III.

**Fig 2:**
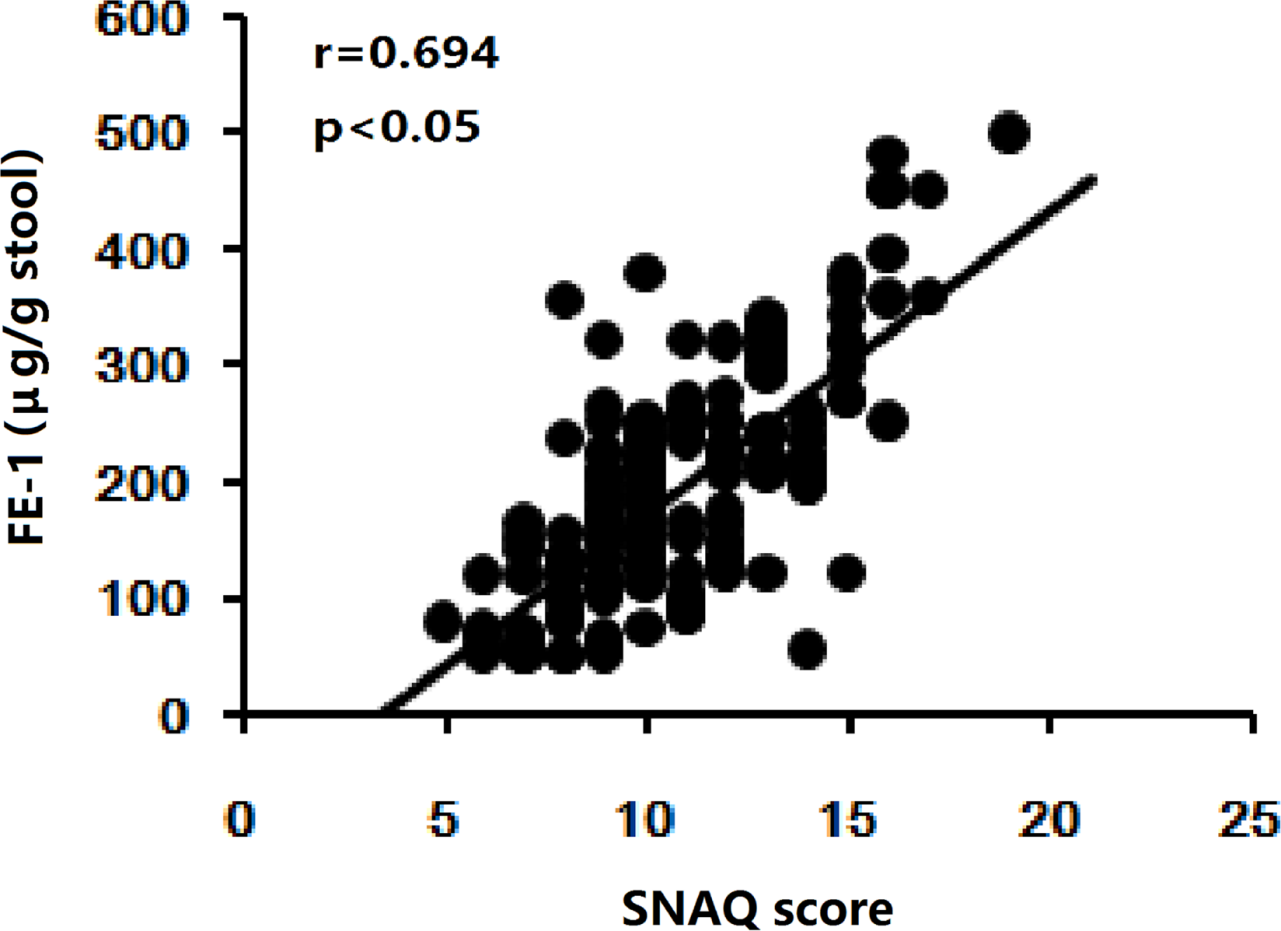
The correlation between SNAQ scores and FE-1 levels was also analyzed in patients with CHF. Spearman coefficient was used for measuring linear correlation between variables. A positive correlation was found between SNAQ scores and FE-1 (r = 0.694, p < 0.001).

PEI was found in patients with CHF by FE-1evaluation and the results of FE-1 levels in all groups were given in Table 2. Though FE-1 levels were not significant different between controls and patients of NYHA I/II, an obvious reduction of FE-1 levels were observed in patients of NYHA III/IV. The lowest content of FE-1 levels was found in patients of NYHA IV. According to the results of FE-1levels in this study, the frequency of severe PEI increased accompanying with the increase of NYHA grading. In this study, the appetite assessment was undergone by SNAQ. The results showed that appetite loss occurred in patients with CHF. Similar to FE-1 levels, the SNAQ scores was reduced gradually, as the NYHA grading increased. The correlation between SNAQ and FE-1 was also analyzed. A positive correlation was found between SNAQ scores and FE-1 (r = 0.694, p < 0.001) (Fig 2).

**Table 2:** The comparison of FE-1 levels and SNAQ scores among the control and patients with CHF.

	Control (n = 20)	NYHA I/II (n = 32)		NYHA III (n = 42)		NYHA IV (n = 30)	
FE-1 (μg/g stool)	276.15±86.76	243.70±85.32	^a^	166.55±93.49	^a^^,^^b^	149.35±76.79	^a^^,^^b^^,^^c^
FE-1 ≥ 200 (μg/g stool) n (%)	0 (0.00%)	24 (75.00%)		13 (30.95%)		8 (26.67%)	
FE-1 ≥ 100 and < 200 (μg/g stool) n (%)	0(0.00%)	7 (21.88%)	^a^	15 (35.71%)	^a^^,^^b^	10 (33.33%)	^a^^,^^b^
FE-1 < 100 (μg/g stool) n (%)	0 (0.00%)	1 (3.13%)		14 (33.33%)		12 (40.00%)	
SNAQ	17.85±1.73	12.77±2.04	^a^	9.82±2.01	^a^^,^^b^	9.61±2.57	^a^^,^^b^^,^^c^
SNAQ ≥14	20 (100.00%)	22 (68.75%)	^a^	9 (21.43%)	^a^^,^^b^	3 (10.00%)	^a^^,^^b^
SNAQ<14	0 (0.00%)	10 (31.25%)		33 (78.57%)		27 (90.00%)	

FE-1: Fecal elastase-1; SNAQ: the simplified nutritional appetite questionnaire; CHF: chronic heart failure; NYHA: New York Heart Association; Values expressed in mean±SD or frequency

^a^*P*<0.05: versus control

^b^*P*<0.05: versus NYHA I/II

^c^*P*<0.05: versus NYHA III.

**Fig 3:**
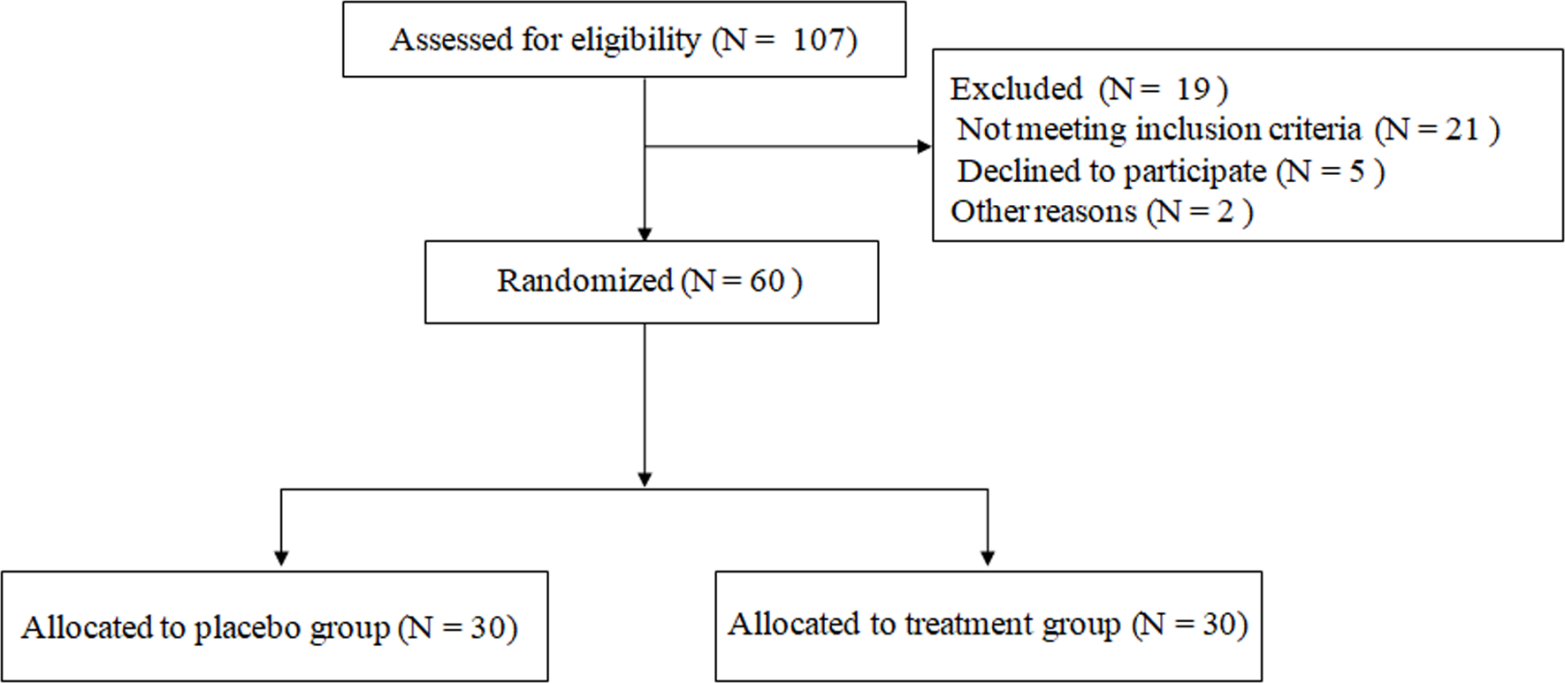
Flow diagram of patients selection in experiment 2.

**Fig 4:**
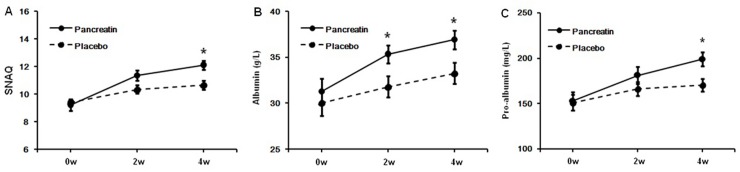
The effect of pancreatin on the appetite loss was evaluated in CHF patients with FE-1 levels <200 μg/g stool and SNAQ < 14. A. SNAQ score; B. Albumin; C. Pro-albumin. Compared with placebo group, pancreatin could significantly improve the SNAQ score in treatment group as well as albumin and pro-albumin. The data are expressed as means ± SD. *p<0.05.

The effect of pancreatin on the appetite loss was evaluated in CHF patients (FE-1 levels <200 μg/g stool and SNAQ < 14) in experiment 2 (Fig 3). 60 patients were randomly divided into two groups (placebo group, N = 30; treatment group, N = 30). The basic characteristics of the enrolled patients were not significantly different between the two groups, including causes, gender distribution, age and CHF severity (S1 Table). After 4-week treatment, pancreatin could significantly improve the appetite loss in treatment group (SNAQ score: placebo group: 0w: 9.40±1.22, 2w: 10.36±1.47, 4w: 10.88±1.59; pancreatin group: 0w: 9.24±2.05, 2w: 11.36±1.87, 4w: 12.12±1.67) and increase albumin and pro-albumin as well (albumin (g/L): placebo group: 0w: 30.04±6.77, 2w: 31.80±5.77, 4w: 33.28±5.68; pancreatin group: 0w: 31.28±7.01, 2w: 35.36±4.77, 4w: 36.92±5.00; pro-albumin (mg/L): placebo group: 0w: 151.24±43.31, 2w: 166.36±38.06, 4w: 170.44±35.88; pancreatin group: 0w: 152.92±51.02, 2w: 181.24±46.66, 4w: 199.04±39.78) (Fig 4). The result indicated that pancreatic enzymes supplement could attenuate the appetite loss in CHF.

## Discussion

Defined as the alteration of pancreatic function, pancreatic exocrine insufficiency (PEI) will lead to maldigestion resulting from the reduction of exocrine secretion in the pancreas, which is caused by diseases of the exocrine pancreas and the endocrine pancreas (e.g. acute pancreatitis, chronic pancreatitis, pancreatic cancer and diabetes mellitus) [17]. Anatomical changes following other gastrointestinal surgery or other digestive tract disorders will also cause the secondary PEI. Malnutrition in the main clinical consequence of PEI represented as maldigestion and poor absorption of nutrients [18]. The impact of PEI on malnutrition in non-pancreatic diseases has also been studied. For instance, changes in pancreatic histology and exocrine function are also commonly reported in chronic renal failure, and exocrine pancreatic insufficiency has been implicated in the pathogenesis of the wasting syndrome of chronic renal failure [19]. Malnutrition is also reported to be one complication of CHF, which may lead to thinning of myofibrils, further deterioration of cardiac contractility and further reduction of cardiac output in turn [20]. Highly prevalent in these patients with CHF, malnutrition is thought to be an important risk factor for morbidity and mortality. According to the previous reports, the test is less invasive and expensive than the current secretin-cholecystokinin test (the gold standard of exocrine pancreatic status), which makes it more convenient for clinical use [21]. Feces has being increasingly applied in clinical tests for gastrointestinal diseases. For example, testing for mutant K-ras in stool has been proposed for the detection of pancreatic and colorectal cancer in old adults and indicates a potential use for early detection of pancreatic cancer. In that study, PEI was determined by FE-1 and showed no significant differences between subjects with or without K-ras mutation [22].FE-1 level has recently been highlighted as an indicator of pancreatic exocrine function, and levels of FE-1 lower than 200 μg/g of stool indicates the existence of PEI (the concentration of 100 to 200 μg/g stool indicating mild to moderate PEI, and the concentration below 100 μg/g stool indicating severe PEI) [23].FE-1 levels have been assessed in pancreatitis, pancreatic cancer, trauma, or chronic renal failure. Obvious decrease of FE-1 levels has been observed in these diseases [24]. In the current study, we also used FE-1 levels as the indicator of PEI, and the reduction of FE-1 levels were found in patients with CHF. The FE-1 levels reduced along with the increasing severity of CHF and the decreasing nutritional parameters (such as BMI, albumin, pro-albumin and hemoglobin), indicating that patients with CHF might have PEI and FE-1 could be used as an index of malnutrition in CHF.

Commonly occurred in chronic diseases, decreased appetite and involuntary weight loss have a negative impact on both quality of life and eventual mortality [25]. Appetite and body weight alter in a wide variety of diseases, including cancer, renal failure, chronic obstructive pulmonary disease and HIV, as well as heart failure [26]. Appetite loss is reported to be associated with a significant higher risk of all-cause mortality and has an independent effect on survival among old people receiving home care. Weight loss induced by appetite loss can be found in all age groups, which constitutes a special problem in older adults. The Council of Nutrition Appetite Questionnaire (CNAQ) and Simplified Nutritional Appetite Questionnaire (SNAQ) are the appetite-monitoring instruments, which is specifically validated for identifying persons at risk of malnutrition and significant weight loss [16, 27].Previous study also shows that SNAQ is a more efficient, reliable, and valid clinical tool, in light of its brevity and comparable reliability, and with appraisal parameters that focus on the singular construct of appetite with a view to preventing weight loss [28, 29]. The total SNAQ score range from 4 to 20. A SNAQ score<14 has been reported to be a significant risk of weight loss>5% within 6 months with a sensitivity of 81.5% and a specificity of 76.4%. Its sensitivity and specificity reach 88.2% and 83.5%, respectively, to predict a >10% weight loss. In our study, the appetite assessment was undergone by SNAQ and showed that appetite loss was commonly seen in patients with CHF. In addition to the abnormality of cardiac and pulmonary function, the congestion of CHF may also cause the functional change in other organs, including liver, kidney, brain and gastrointestinal tract, which probably causes the appetite loss [30]. However, the relationship between CHF and pancreatic function is rarely reported. Therefore, it is rational to be hypothesized that CHF-induced anorexia is probably not only caused by gastrointestinal congestion, but also by PEI. In the present study, a positive correlation was found between SNAQ scores and FE-1 in patients with CHF, supporting the hypothesis that PEI might have a potential effect on the CHF induced appetite loss.

The pancreas is the first organ that decreases its quality of exocrine excretion when there is inflammation and malnutrition. While, in other inflamed organs, such as the heart, many inflammatory mediators are overproduced, inducing anorexia directly on the hypothalamus. In our study, the obvious changes of TNF-α, IL-1β, IL-6 and leptin were found, which are reported to be involved in diseases-induced appetite loss [31–34]. Serum concentrations of TNF-α, IL-1β and IL-6 were increased along with the severity of heart failure and the decrease of SNAQ scores as well. These pro-inflammatory cytokines may directly regulate the appetite. On the other hand, they could also influence on the pancreatic exocrine secretion by inducing a chronic inflammation on the pancreas, since they are proven as important mediators in the pathogenesis of pancreatitis. Leptin is expressed almost exclusively by adipocytes and its production is influenced by hormones, cytokines and nutrients. Leptin is involved in the starvation signal, when low, it prompts increased appetite and decreased energy expenditure. Adipocytes increase leptin expression when cell size increases, which will result in depresses appetite and increased energy expenditure. Leptin deficiency also exists due to excessive thinness, such as results from starvation, weight loss, anorexia or cancer-induced cachexia [35,36]. In our study, serum leptin was reduced along with the NYHA grading, indicating its possible role in heart failure-associated appetite loss.

Oral pancreatic enzyme replacement therapy (PERT) is the treatment of choice for PEI, together with nutritional management and specific nutritional supplementation if required [37].Pancreatin (Creon 10000^®^) is a commercial mixture of several digestive enzymes produced by the exocrine cells of the pancreas, which is composed of amylase, lipase and protease. Taken by mouth, it is clinically used to treat PEI [38]. For further detecting the effect of PEI on the appetite loss in CHF, pancreatin was applied in patients with CHF in our study. Our result showed that PERT could attenuate the HF-induced appetite loss, further indicating that PEI might play an aggravating role in HF-induced appetite loss.

In conclusion, appetite loss is commonly found in chronic heart failure, and is partially associated with pancreatic exocrine insufficiency. Oral pancreatic enzyme replacement therapy attenuates the chronic heart failure-induced appetite loss. These results suggest a potential pancreatic-cardiac relationship in chronic heart failure, and further experiment is needed for clarifying the possible mechanisms.

## Supporting information

S1 Table The basic characteristics of the enrolled patients in experiment 2. (DOCX)

## References

[1] Gandhi PU, Pinney S. Management of ChronicHeart Failure: Biomarkers, Monitors, and Disease Management Programs.Ann Glob Health. 2014–;80(1):46–54. doi: 10.1016/j.aogh.2013.12.005 24751564 10.1016/j.aogh.2013.12.005

[2] Irving G, Holden J, Edwards J, Reeve J, Dowrick C, Lloyd-Williams M. Chronicheart failure guidelines: do they adequately address patient need at the end-of-life? Int J Cardiol;168(3):2304–9. doi: 10.1016/j.ijcard.2013.01.189 23465240 10.1016/j.ijcard.2013.01.189

[3] McMurray JJ, Pfeffer MA. Heart failure. Lancet 2005; 365 (9474): 1877–89. doi: 10.1016/S0140-6736(05)66621-4 15924986 10.1016/S0140-6736(05)66621-4

[4] Chronic Heart Failure: National Clinical Guideline for Diagnosis and Management in Primary and Secondary Care: Partial Update. National Clinical Guideline Centre 2010: 38–70. 22741186

[5] Dickstein K, Cohen-Solal A, Filippatos G, McMurray JJ, Ponikowski P, Poole-Wilson PA, et al ESC Guidelines for the diagnosis and treatment of acute and chronic heart failure 2008: the Task Force for the Diagnosis and Treatment of Acute and Chronic Heart Failure 2008 of the European Society of Cardiology. Developed in collaboration with the Heart Failure Association of the ESC (HFA) and endorsed by the European Society of Intensive Care Medicine (ESICM). Eur. Heart J 2008; 29: 2388–442. doi: 10.1093/eurheartj/ehn309 18799522 10.1093/eurheartj/ehn309

[6] Stevenson LW, Pande R. Witness to progress. Circ Heart Fail. 2011;4:390–2. doi: 10.1161/CIRCHEARTFAILURE.111.963066 21772014 10.1161/CIRCHEARTFAILURE.111.963066

[7] Cubbon RM, Gale CP, Kearney LC, Schechter CB, Brooksby WP, Nolan J, F, et al Changing characteristics and mode of death associated with chronic heart failure caused by left ventricular systolic dysfunction: a study across therapeutic eras. Circ Heart Fail. 2011;4:396–403. doi: 10.1161/CIRCHEARTFAILURE.110.959882 21562056 10.1161/CIRCHEARTFAILURE.110.959882

[8] Ross JS, Chen J, Lin Z, Bueno H, Curtis JP, Keenan PS, Normand SL, et al Recent national trends in readmission rates after heart failure hospitalization. Circ Heart Fail. 2010;3:97–103. doi: 10.1161/CIRCHEARTFAILURE.109.885210 19903931 10.1161/CIRCHEARTFAILURE.109.885210 PMC2830811

[9] Meijers WC, Januzzi JL, deFilippi C, Adourian AS, Shah SJ, van Veldhuisen DJ, et al Elevated plasma galectin-3 is associated with near-term rehospitalization in heart failure: a pooled analysis of 3clinical trials. Am Heart J. 2014;167(6):853–60.e4. doi: 10.1016/j.ahj.2014.02.011 24890535 10.1016/j.ahj.2014.02.011

[10] Landi F, Calvani R, Tosato M, Martone AM, Ortolani E, Savera G, et al Anorexiaof Aging: Risk Factors, Consequences, and Potential Treatments.Nutrients. 2016;8(2):69 doi: 10.3390/nu8020069 26828516 10.3390/nu8020069 PMC4772033

[11] Rahman A, Jafry S, Jeejeebhoy K, Nagpal AD, Pisani B, Agarwala R. Malnutrition and Cachexia in Heart Failure. JPEN J Parenter Enteral Nutr. 2016;40(4):475–86. doi: 10.1177/0148607114566854 25634161 10.1177/0148607114566854

[12] Kemp CD, Conte JV. The pathophysiology ofheart failure.Cardiovasc Pathol. 2012;21(5):365–71. doi: 10.1016/j.carpath.2011.11.007 22227365 10.1016/j.carpath.2011.11.007

[13] Özcan M, Öztürk GZ, Köse M, Emet S, Aydın Ş, Arslan K, et al Evaluation of malnutrition with blood ghrelin and fecal elastase levels in acute decompensated heart failure patients.Turk Kardiyol Dern Ars. 2015;43(2):131–7. doi: 10.5543/tkda.2015.06606 25782117 10.5543/tkda.2015.06606

[14] Hunt SA; American College of Cardiology; American Heart Association Task Force on Practice Guidelines (Writing Committee to Update the 2001 Guidelines for the Evaluation and Management of Heart Failure). ACC/AHA 2005 guideline update for the diagnosis and management of chronic heart failure in the adult: a report of the American College of Cardiology/American Heart Association Task Force on Practice Guidelines (Writing Committee to Update the 2001 Guidelines for the Evaluation and Management of Heart Failure). J Am Coll Cardiol. 2005;46(6):e1–82. doi: 10.1016/j.jacc.2005.08.022 16168273 10.1016/j.jacc.2005.08.022

[15] Suresh KP. An overview of randomization techniques: An unbiased assessment of outcome in clinical research. J Hum Reprod Sci. 2011; 4(1): 8–11. doi: 10.4103/0974-1208.82352 21772732 10.4103/0974-1208.82352 PMC3136079

[16] Wilson MM, Thomas DR, Rubenstein LZ, Chibnall JT, Anderson S, Baxi A, et al Appetite assessment: simple appetite questionnaire predicts weight loss in community-dwelling adults and nursing home residents. Am J Clin Nutr. 2005;82(5):1074–81. 16280441 10.1093/ajcn/82.5.1074

[17] Lindkvist B. Diagnosis and treatment of pancreatic exocrine insufficiency.World J Gastroenterol. 2013;19(42):7258–66. doi: 10.3748/wjg.v19.i42.7258 24259956 10.3748/wjg.v19.i42.7258 PMC3831207

[18] Pongprasobchai S. Maldigestion from pancreatic exocrine insufficiency. J Gastroenterol Hepatol. 2013;28 Suppl 4:99–102. 10.1111/jgh.12406 24251713

[19] Aguilera A, Bajo MA, Espinoza M, Olveira A, Paiva AM, Codoceo R, et al Gastrointestinal and pancreatic function in peritoneal dialysis patients: their relationship with malnutrition and peritoneal membrane abnormalities. Am J Kidney Dis. 2003;42(4):787–96. 14520630 10.1016/s0272-6386(03)00920-x

[20] Saitoh M, Rodrigues Dos Santos M, von Haehling S. Muscle wasting inheart failure: The role of nutrition.Wien Klin Wochenschr. 2016;128(Suppl 7):455–465. doi: 10.1007/s00508-016-1100-z 27761739 10.1007/s00508-016-1100-z

[21] Leeds JS, Oppong K, Sanders DS. The role of fecal elastase-1in detecting exocrine pancreatic disease. Nat Rev Gastroenterol Hepatol. 2011;;8(7):405–15. doi: 10.1038/nrgastro.2011.91 21629239 10.1038/nrgastro.2011.91

[22] Haug U, Hillebrand T, Bendzko P, Löw M, Rothenbacher D, Stegmaier C, et al Mutant-enriched PCR and allele-specific hybridization reaction to detect K-ras mutations in stool DNA: high prevalence in a large sample of older adults. Clin Chem.2007;53(4):787–90. doi: 10.1373/clinchem.2006.078188 17317884 10.1373/clinchem.2006.078188

[23] Xu Y, Wu D, Zeng Y, Wang X. Pancreatic exocrine function and morphology following an episode of acute pancreatitis. Pancreas. 2012;41(6):922–7. doi: 10.1097/MPA.0b013e31823d7f2d 22481293 10.1097/MPA.0b013e31823d7f2d

[24] Dominici R, Franzini C. Fecalelastase-1 as a test for pancreatic function: a review. Clin Chem Lab Med. 2002r;40(4):325–32. doi: 10.1515/CCLM.2002.051 12059069 10.1515/CCLM.2002.051

[25] Loncar G, Springer J, Anker M, Doehner W, Lainscak M, et al Cardiac cachexia: hic et nunc: "hic et nunc"—here and now. Int J Cardiol. 2015;201:e1–12. doi: 10.1016/j.ijcard.2015.10.115 26545926 10.1016/j.ijcard.2015.10.115

[26] Visvanathan R, Chapman IM. Undernutrition and anorexia in the older person. Gastroenterol Clin North Am. 2009;38(3):393–409. doi: 10.1016/j.gtc.2009.06.009 19699404 10.1016/j.gtc.2009.06.009

[27] Wilson MM. Assessment of appetite and weight loss syndromes in nursing home residents. Mo Med.2007;104(1):46–51. 17410825

[28] Phillips MB, Foley AL, Barnard R, Isenring EA, Miller MD.Nutritional screening in community-dwelling older adults: a systematic literature review. Asia Pac J Clin Nutr.2010;19(3):440–9. 20805090

[29] Kaur S, Miller MD, Halbert J, Giles LC, Crotty M. Nutritional status of adults participating in ambulatory rehabilitation. Asia Pac J Clin Nutr.2008;17(2):199–207. 18586637

[30] Rubio Gracia J, Sánchez Marteles M, Pérez Calvo JI. Involvement of systemic venous congestion in heart failure. Rev Clin Esp. 2017;217(3):161–169. doi: 10.1016/j.rce.2016.10.012 27979306 10.1016/j.rce.2016.10.012

[31] Molfino A, Iannace A, Colaiacomo MC, Farcomeni A, Emiliani A, Gualdi G, et al Cancer anorexia: hypothalamic activity and its association with inflammation and appetite-regulating peptides in lung cancer. J Cachexia Sarcopenia Muscle. 2017;8(1):40–47. doi: 10.1002/jcsm.12156 27897393 10.1002/jcsm.12156 PMC5326827

[32] Scheede-Bergdahl C, Watt HL, Trutschnigg B, Kilgour RD, Haggarty A, Lucar E, et al Is IL-6 the best pro-inflammatory biomarker of clinical outcomes of cancer cachexia? Clin Nutr. 2012;31(1):85–8. doi: 10.1016/j.clnu.2011.07.010 21855185 10.1016/j.clnu.2011.07.010

[33] Turrin NP, Ilyin SE, Gayle DA, Plata-Salamán CR, Ramos EJ, Laviano A, et al Interleukin-1beta system in anorectic catabolic tumor-bearing rats. Curr Opin Clin Nutr Metab Care.2004,7(4):419–26. 15192445 10.1097/01.mco.0000134373.16557.92

[34] Barrett GL, Naim T, Trieu J. Leptin-derived peptides that stimulate food intake and increase body weight following peripheral administration. Regul Pept.2011170(1–3):24–30. 10.1016/j.regpep.2011.05.004 21609735

[35] Smiechowska J, Utech A, Taffet G, Hayes T, Marcelli M, Garcia JM. Adipokines in patients with cancer anorexia and cachexia. J Investig Med,2010 58(3):554–9. doi: 10.231/JIM.0b013e3181cf91ca 20215915 10.231/JIM.0b013e3181cf91ca

[36] Hubbard RE, O'Mahony MS, Calver BL, Woodhouse KW. Nutrition, inflammation, and leptin levels in aging and frailty. J Am Geriatr Soc,2008;56(2):279–84. doi: 10.1111/j.1532-5415.2007.01548.x 18179487 10.1111/j.1532-5415.2007.01548.x

[37] Domínguez-Muñoz JE. Pancreaticenzyme replacement therapy for pancreatic exocrine insufficiency: when is it indicated, what is the goal and how to do it? Adv Med Sci. 2011; 56(1):1–5. doi: 10.2478/v10039-011-0005-3 21450558 10.2478/v10039-011-0005-3

[38] Whitcomb DC, Bodhani A, Beckmann K, Sander-Struckmeier S, Liu S, Fuldeore M, et al Efficacy and Safety of Pancrelipase/Pancreatin in Patients With Exocrine Pancreatic Insufficiency and a Medical History of Diabetes Mellitus. Pancreas. 2016;45(5):679–86. doi: 10.1097/MPA.0000000000000514 26495784 10.1097/MPA.0000000000000514

