# Supplementary material for: Pancreatic exocrine insufficiency in patients with chronic heart failure and its possible association with appetite loss
Source: PLoS One. 2017 Nov 20;12(11):e0187804. doi: 10.1371/journal.pone.0187804 (PMC5695817; doi:10.1371/journal.pone.0187804)
Supplement: S1 Table — (DOCX) [file pone.0187804.s001.docx]

| Table S1. The basic characteristics of the enrolled patients in experiment 2. | | | |
| --- | --- | --- | --- |
|  | Placebo group  (N=30) | Treatment group  (N-=30) | *P*-value |
| Men, n(%) | 18 (60.00%) | 20 (66.67%) | NS |
| Age (y) | 70.20±11.48 | 67.63±8.81 | NS |
| BMI, kg/m^2^ | 22.06±3.04 | 22.52±4.13 | NS |
| CHF causes, n (%) |  |  |  |
| Hypertensive heart diseases | 19 | 22 | NS |
| Ischemic heart diseases | 8 | 7 |  |
| Dilated cardiomyopathy | 1 | 0 |  |
| Valvular diseases | 1 | 1 |  |
| Others | 1 | 0 |  |
| SBP (mmHg) | 121.40±19.18 | 126.27±14.40 | NS |
| DBP (mmHg) | 74.23±11.70 | 72.77±10.81 | NS |
| Heart rate (bpm) | 75.20±11.31 | 73.27±9.28 | NS |
| Diabetes, n (%) | 0 | 0 | NS |
| Smoking, n (%) | 0 | 0 | NS |
| Alcohol, n (%) | 0 | 0 | NS |
| NYHA grading |  |  |  |
| NYHA I/II | 9 | 6 | NS |
| NYHA III | 16 | 17 |  |
| NYHA IV | 5 | 7 |  |
| Ejection fraction (%) | 44.77±10.62 | 47.38±13.36 | NS |
| NT-proBNP (pg/ml) | 654.40±268.41 | 662.44±169.75 | NS |
| SNAQ | 9.40±1.22 | 9.24±2.05 | NS |
| FE-1 (μg/g stool) | 114.99±43.57 | 113.95±23.16 | NS |
| CHF: chronic heart failure; NYHA: New York Heart Association; BMI: body mass index; SBP: systolic blood pressure; DBP: diastolic blood pressure; NT-proBNP: N-terminal pro-B type natriuretic peptide; SNAQ: the simplified nutritional appetite questionnaire; FE-1: pancreatic fecal elastase-1; NS: no statistical significance. | | | |
